# Urinary Concentrations of (+)-Catechin and (-)-Epicatechin as Biomarkers of Dietary Intake of Flavan-3-ols in the European Prospective Investigation into Cancer and Nutrition (EPIC) Study

**DOI:** 10.3390/nu13114157

**Published:** 2021-11-20

**Authors:** Enrique Almanza-Aguilera, Daniela Ceballos-Sánchez, David Achaintre, Joseph A Rothwell, Nasser Laouali, Gianluca Severi, Verena Katzke, Theron Johnson, Matthias B Schulze, Domenico Palli, Giuliana Gargano, Maria Santucci de Magistris, Rosario Tumino, Carlotta Sacerdote, Augustin Scalbert, Raul Zamora-Ros

**Affiliations:** 1 Unit of Nutrition and Cancer, Cancer Epidemiology Research Program, Catalan Institute of Oncology (ICO), Bellvitge Biomedical Research Institute (IDIBELL), 08908 Barcelona, Spain; ealmanza@idibell.cat (E.A.-A.); daniela.ceballossanchez@estudiants.urv.cat (D.C.-S.); 2 Nutrition and Metabolism Branch, International Agency for Research on Cancer (IARC-WHO), 69372 Lyon, France; achaintred@iarc.fr (D.A.); ScalbertA@iarc.fr (A.S.); 3 UVSQ, Inserm, CESP U1018, “Exposome and Heredity” Team, Université Paris-Saclay, Gustave Roussy, 94800 Villejuif, France; joseph.rothwell@gustaveroussy.fr (J.A.R.); nasser.laouali@inserm.fr (N.L.); gianluca.severi@inserm.fr (G.S.); 4 Department of Statistics, Computer Science, Applications “G. Parenti” (DISIA), University of Florence, 50121 Florence, Italy; 5 Division of Cancer Epidemiology, German Cancer Research Center (DKFZ), 69120 Heidelberg, Germany; v.katzke@dkfz-heidelberg.de (V.K.); t.johnson@dkfz-heidelberg.de (T.J.); 6 Department of Molecular Epidemiology, German Institute of Human Nutrition Potsdam-Rehbruecke, 14558 Nuthetal, Germany; mschulze@dife.de; 7 Institute of Nutritional Science, University of Potsdam, 14469 Potsdam, Germany; 8 Cancer Risk Factors and Life-Style Epidemiology Unit, Institute for Cancer Research, Prevention and Clinical Network (ISPRO), 50139 Florence, Italy; d.palli@ispro.toscana.it; 9 Epidemiology and Prevention Unit, Fondazione IRCCS Istituto Nazionale dei Tumori, 20133 Milan, Italy; giuliana.gargano@istitutotumori.mi.it; 10 Dipartimento di Medicina Clinica e Chirurgia, Federico II University, 80131 Naples, Italy; masantuc@unina.it; 11 Cancer Registry and Histopathology Department, Provincial Health Authority (ASP 7), 97100 Ragusa, Italy; rtuminomail@gmail.com; 12 Unit of Cancer Epidemiology, Città della Salute e della Scienza University-Hospital, 10126 Turin, Italy; carlotta.sacerdote@cpo.it

**Keywords:** urine, catechin, epicatechin, flavan-3-ols, biomarkers, intake, EPIC

## Abstract

This study examines the correlation of acute and habitual dietary intake of flavan-3-ol monomers, proanthocyanidins, theaflavins, and their main food sources with the urinary concentrations of (+)-catechin and (-)-epicatechin in the European Prospective Investigation into Cancer and Nutrition study (EPIC). Participants (N = 419, men and women) provided 24-h urine samples and completed a 24-h dietary recall (24-HDR) on the same day. Acute and habitual dietary data were collected using a standardized 24-HDR software and a validated dietary questionnaire, respectively. Intake of flavan-3-ols was estimated using the Phenol-Explorer database. Concentrations of (+)-catechin and (-)-epicatechin in 24-h urine were analyzed using tandem mass spectrometry after enzymatic deconjugation. Simple and partial Spearman’s correlations showed that urinary concentrations of (+)-catechin, (-)-epicatechin and their sum were more strongly correlated with acute than with habitual intake of individual and total monomers (acute *r*_partial_ = 0.13–0.54, *p* < 0.05; and habitual *r*_partial_ = 0.14–0.28, *p* < 0.01), proanthocyanidins (acute *r*_partial_ = 0.24–0.49, *p* < 0.001; and habitual *r*_partial_ = 0.10–0.15, *p* < 0.05), theaflavins (acute *r*_partial_ = 0.22–0.31, *p* < 0.001; and habitual *r*_partial_ = 0.20–0.26, *p* < 0.01), and total flavan-3-ols (acute *r*_partial_ = 0.40–0.48, *p* < 0.001; and habitual *r*_partial_ = 0.23–0.33, *p* < 0.001). Similarly, urinary concentrations of flavan-3-ols were weakly correlated with both acute (*r*_partial_ = 0.12–0.30, *p* < 0.05) and habitual intake (*r*_partial_ = 0.10–0.27, *p* < 0.05) of apple and pear, stone fruits, berries, chocolate and chocolate products, cakes and pastries, tea, herbal tea, wine, red wine, and beer and cider. Moreover, all comparable correlations were stronger for urinary (-)-epicatechin than for (+)-catechin. In conclusion, our data support the use of urinary concentrations of (+)-catechin and (-)-epicatechin, especially as short-term nutritional biomarkers of dietary catechin, epicatechin and total flavan-3-ol monomers.

## 1. Introduction

Flavan-3-ols or flavanols are a large and complex flavonoid subclass widely present in a number of plant-origin foods such as pome fruits (e.g., apples and pears), legumes, cocoa, tea and wine [1,2]. Flavan-3-ols can be divided into monomers: catechin, epicatechin, epigallocatechin, gallocatechin, and their gallate derivatives; and their oligomeric and polymeric forms, also known as proanthocyanidins, of which the degree of polymerization can range from 2 to 50 units or more [3,4]. Theaflavins and thearubigins are flavanol-derived compounds formed as result of oxidation and polymerization reactions during fermentation of the green leaves in black tea production [5]. Bioavailability of flavan-3-ols depends largely on their degree of polymerization. While monomers are partially absorbed in the small intestine; oligomers and polymers need to be biotransformed by the colonic microbiota to low molecular weight metabolites (phenolic acids and lactones) prior to absorption [6].

Flavan-3-ols are the most consumed flavonoid class by far in Europe and globally, contributing to >70% of total flavonoids [7]. In European adults, flavan-3-ol intake varies from 124.8–160.5 mg/day (for Greek women and men) to 376.6–453.6 mg/day (for UK women and men), flavan-3-ol monomers (18.6–44.9%) and proanthocyanidins (48.8–80.8%) being the main contributors [2]. Flavan-3-ols have been reported to exhibit antioxidant, anti-inflammatory, immunomodulatory, antiallergic, and antiviral effects, as well as to have the capacity to modulate gut microbiome [8]. In addition, epidemiological studies have suggested that the intake of flavan-3-ols may contribute to the prevention of several chronic diseases such as diabetes, metabolic syndrome, cardiovascular disease and some cancer types [9,10].

Current epidemiological data on flavan-3-ol intake mostly rely on self-reported questionnaires, including 24-h dietary recalls (24-HDR), food records, and food frequency questionnaires (FFQ), which estimate flavan-3-ol exposure using food composition databases [1,11]. However, although these instruments can clearly differentiate between extreme intakes; they do not take into account the variability of food composition, as well as the extensive metabolism that flavan-3-ols undergo after their intake. In addition, traditional methods can be hampered by the individual’s misreporting of their consumption [12]. To overcome such limitations, over the past decades, there has been an increasing interest in the identification and quantification of small molecules present in blood and urine reflecting the intake of specific foods or food components, including polyphenols [13]. Nutritional biomarkers are essential to accurately estimate the intake of polyphenols and properly investigate their potential beneficial relationships with health outcomes. However, to date, there is limited evidence of potential flavan-3-ols intake biomarkers. Thus, in the current study, we aimed to assess the correlation between acute and habitual dietary intake of flavan-3-ols, their main food sources and the 24-h urinary concentrations of two flavan-3-ol monomers: (+)-catechin and (-)-epicatechin. The rationale to include proanthocyanidins and theaflavins in our study is that both usually concurrent with monomers in some flavanol-rich food sources such as apples, pears and tea.

## 2. Materials and Methods

### 2.1. Study Population

The European Prospective Investigation into Cancer and Nutrition (EPIC) study is a prospective study, which involves the participation of 23 centers from 10 European countries. It includes more than half a million adults of both sexes, mostly recruited from the general population between 1992 and 2000 [14,15]. The investigation was approved by the ethical review boards of the International Agency for Research on Cancer (IARC) and all the local institutions involved. All participants also signed an informed consent form. In the present study, a convenience subsample of 419 women and men, aged between 34 and 73 years from eight centers, in three countries (France, Italy and Germany) was included. It should be noted that in France and Naples (Italy) only women were recruited. All participants completed a single 24-h dietary recall (24-HDR) and provided a 24-h urine sample on the same day.

### 2.2. Dietary and Lifestyle Information

Acute dietary intake was assessed by means of a single 24-HDR, applied in a face-to-face interview using the standardized software EPIC-Soft (renamed as GloboDiet) [16]. Habitual dietary data of the previous year were collected using a quantitative dietary questionnaire (DQ) specific to each center in France, Germany, and Italy, except in Naples where a semi-quantitative food frequency questionnaire was applied [14,15]. The DQs were developed and validated in each center [15]. The average time interval between the application of the DQ and the 24-HDR interview varied between 1 to 3 years, according to each country/center [17]. Dietary intakes of three subclasses of flavan-3-ols, including monomers, proanthocyanidins and theaflavins, were estimated using the Phenol-Explorer database [11,18]. Total intake of each flavan-3-ol subclass and total intake of flavan-3-ols were calculated as the sum of the individual compounds belonging to each subclass. Total daily energy intake was estimated using the EPIC standardized nutrient database [19]. The acute and habitual intakes of 14 food and food groups (i.e., apple and pear, grape, berries, banana, chocolate and chocolate products, cakes and pastries, coffee, tea, herbal tea, wine, red wine, beer and cider) were used to calculate correlations with urinary (+)-catechin and (-)-epicatechin. The selection of these foods was based on their contribution (≥0.2%) to the intake of flavan-3-ol monomers in Mediterranean and non-Mediterranean countries from the EPIC study [2]. Lifestyle information such as smoking status and physical activity was obtained at the beginning of the study by means of standardized questionnaires [14]. Demographic and anthropometric data were self-reported by study participants during 24-HDR interviews.

### 2.3. Samples and Analytical Method

Urine samples were collected, stored, and analyzed as previously described [20]. Briefly, urine samples were collected over a 24-h period and stored at −20 °C using boric acid as a preservative. The integrity of the 24-h urine samples was monitored using *p*-aminobenzoic acid. Samples with *p*-aminobenzoic acid recovery <70 and >110% were excluded from this study. (+)-Catechin and (-)-epicatechin were analyzed in 24-h urine samples using an ultra-performance liquid chromatography–tandem mass spectrometry system (UPLC-MS/MS). An Acquity CSH C18 column (Waters, 2.1 mm × 100 mm, 1.7 μm) maintained at 50 °C, was used as UPLC stationary phase, whereas the following solvents were used as mobile phase: solvent A, 0.1% formic acid in methanol/water 10/90 (*v*/*v*/*v*); solvent B, acetonitrile. Before their analysis, urine samples were treated with a β-glucuronidase/sulfatase enzyme mixture and extracted twice with ethyl acetate. All phenolic groups in (+)-catechin and (-)-epicatechin were quantitatively marked using a differential isotope-labelling method. The limits of quantification (LOQ) for (+)-catechin and (-)-epicatechin were 0.04 and 0.10 μM, respectively. Intra-assay and inter-assay coefficient of variability (CV) were 7.5 and 17.4 for (+)-catechin, and 6.0 and 17.5 for (-)-epicatechin. Urinary excretion of both (+)-catechin and (-)-epicatechin was expressed as μmol/24 h. Total urinary flavan-3-ols was calculated as the sum of (+)-catechin and (-)-epicatechin.

### 2.4. Statistical Analyses

Urinary concentrations of (+)-catechin and (-)-epicatechin that fell below the LOQ were established to values corresponding to half of the LOQ. Descriptive statistics, including number of non-consumers or number of samples <LOQ, median and 10th and 90th percentiles were used for both urinary concentrations and dietary intakes of flavan-3-ols. The Kruskal-Wallis test was used to compare the levels of urinary flavan-3-ols by demographic and lifestyle characteristics. Spearman’s rank correlations were used to assess the relationships between urinary flavan-3-ol concentrations and dietary variables (i.e., flavan-3-ols and food sources) estimated using the 24-HDR and DQ. Partial Spearman’s correlations were conducted to assess the correlation between dietary flavan-3-ol intake and urinary flavan-3-ol levels while adjusting for potential confounders, including BMI, age at recruitment, sex, center, smoking status (i.e., never, former, current smoker) and total energy intake (obtained from the 24-HDR or DQ, as appropriate). All analyses were conducted using SPSS software version 25.0 (IBM Corp. Released 2017. IBM SPSS Statistics for Windows, Version 25.0. Armonk, NY, USA: IBM Corp.). The statistical significance level was set to *p* < 0.05. To illustrate the above mentioned correlations, we used the “ggcorrplot” r-package within the RStudio software version 1.4.1717.

## 3. Results

### 3.1. Urinary Flavanol Concentrations

Median 24-h urinary excretions of (+)-catechin and (-)-epicatechin, and their sum, according to sociodemographic and lifestyle characteristics are shown in Table 1. Of the 419 participants in the current study, 22 and 18 had urinary concentrations of (+)-catechin and (-)-epicatechin below the LOQ, respectively. Urinary concentrations of (-)-epicatechin were higher than (+)-catechin in all centers and in all categories related to sociodemographic and lifestyle variables. The highest median urinary concentrations for (+)-catechin and (-)-epicatechin were observed in Heidelberg (Germany): 0.15 and 0.29 μmol/24 h, respectively; whereas the lowest concentrations were observed in Naples (Italy): 0.06 and 0.17 μmol/24 h, respectively. Moreover, the highest urinary excretion of total flavan-3-ol was observed in Paris and Turin (0.45 μmol/24 h), and the lowest in Naples (0.20 μmol/24 h). Excretions of (+)-catechin, (-)-epicatechin, and their sum were higher in men than in women. There were no substantial differences in urinary concentrations of (+)-catechin, (-)-epicatechin, and their sum among categories of age, BMI, smoking status, and total energy intake.

### 3.2. Intake of Flavan-3-ols and Food Sources

Median intake of dietary flavan-3-ols and their main food sources are shown in Table 2. The acute intake of total flavan-3-ols, and total monomers and proanthocyanidins was higher than their corresponding habitual intake. Conversely, habitual intakes of eight individual flavan-3-ol monomers (i.e., (+)-catechin 3-O-gallate, (+)-catechin 3-O-glucose, (+)-gallocatechin, (+)-gallocatechin 3-O-gallate, (-)-epicatechin 3-O-gallate, (-)-epigallocatechin, (-)-epigallocatechin 3-O-gallate, and (-)-epicatechin-(2a-7)(4a-8)-epicatechin 3-O-galactoside), as well as of individual and total theaflavins were higher than their respective acute intakes. Intake of (+)-catechin and (-)-epicatechin in the 24-HDR (29.8 and 50.5 mg/day) was higher than their intake in the DQ (14.0 and 18.1 mg/day). In both 24-HDR and DQ, (-)-epicatechin compounds were consumed more than (+)-catechin compounds. Regarding food sources of flavan-3-ols, coffee was the food most consumed in both 24-HDR and DQ, with a median intake of 175 and 150 g/day, respectively.

### 3.3. Correlations between Urinary and Dietary Flavan-3-ols

**Figure 1:**
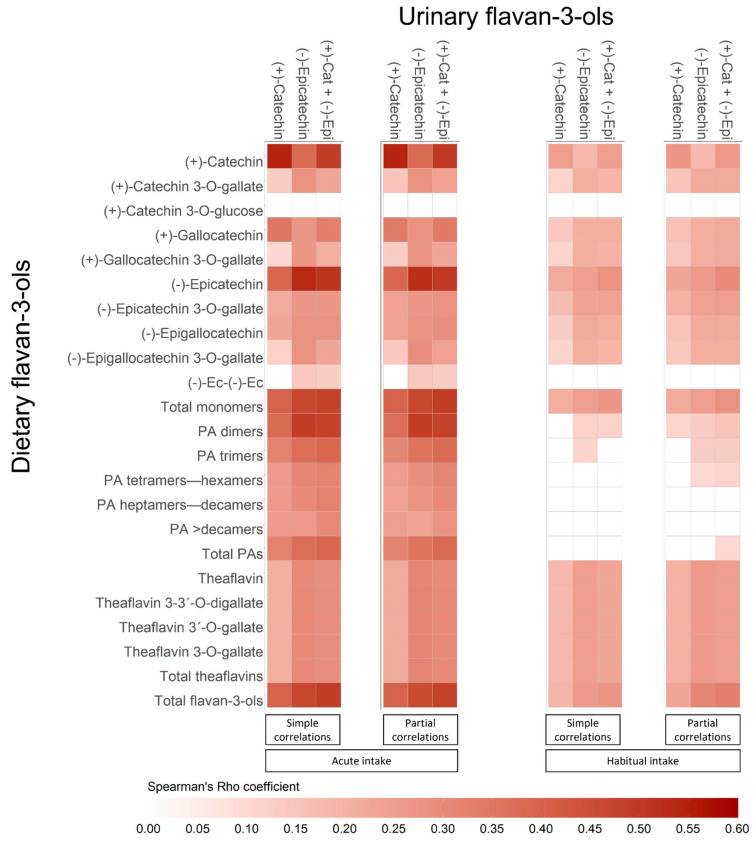
Heatmaps showing simple and partial Spearman’s correlations between urinary and dietary intake (acute and habitual) of flavan-3-ols. Abbreviations: 24-HDR, 24-h dietary recall; (+)-Cat, catechin; DQ, dietary questionnaire; Ec-Ec, (-)-epicatechin-(2a-7)(4a-8)-epicatechin 3-O-galactoside; (-)-Epi, (-)-epicatechin; PA, proanthocyanidins.

Simple and partial Spearman’s correlations between urinary concentrations of (+)-catechin, (-)-epicatechin, and their sum, and the acute and habitual intake of all flavan-3-ols are illustrated in Figure 1. Urinary (+)-catechin and (-)-epicatechin positively correlated with their acute (*r* = 0.53–0.54, *p* < 0.001) and habitual intake (*r* = 0.25, *p* < 0.001). Both acute and habitual intake of individual and total flavan-3-ol monomers (i.e., (+)-catechin and (-)-epicatechin compounds) correlated with urinary concentrations of (+)-catechin (*r* = 0.11–0.40, *p* < 0.05), (-)-epicatechin (*r* = 0.14–0.48, *p* < 0.01), and their sum (*r* = 0.13–0.48, *p* < 0.05). No relation was observed with (+)-catechin-3-O-glucose intake. Acute intake of total and individual proanthocyanidins correlated with urinary (+)-catechin (*r* = 0.26–0.38, *p* < 0.001), (-)-epicatechin (*r* = 0.26–0.49, *p* < 0.001) and their sum (*r* = 0.30–0.48, *p* < 0.001). Habitual intake of proanthocyanidin dimers and trimers mainly correlated with urinary (-)-epicatechin (*r* ~0.12, *p* < 0.05). Both, acute and habitual intake of total and individual theaflavins correlated with urinary (+)-catechin (*r* = 0.18–0.21, *p* < 0.01), (-)-epicatechin (*r* = 0.25–0.30, *p* < 0.001), and their sum (*r* = 0.24–0.29, *p* < 0.001).

Results of partial Spearman’s correlations between urinary and dietary flavan-3-ols were relatively similar to simple correlations (Figure 1). Contrary to simple correlations, partial ones showed that urinary (+)-catechin concentrations, and their sum with urinary (-)-epicatechin, correlated slightly with the habitual intakes of proanthocyanidin dimers (*r*_partial_ = 0.11, *p* < 0.05), trimers and tetramers-hexamers (*r*_partial_ = 0.11–0.13, *p* < 0.05), respectively. Similarly, in partial correlations the sum of urinary (+)-catechin and (-)-epicatechin was weak but significantly correlated with the habitual intake of total proanthocyanidins (*r*_partial_ = 0.10, *p* < 0.05).

### 3.4. Correlations between Urinary Flavan-3-ols and Food Source Intake

**Figure 2:**
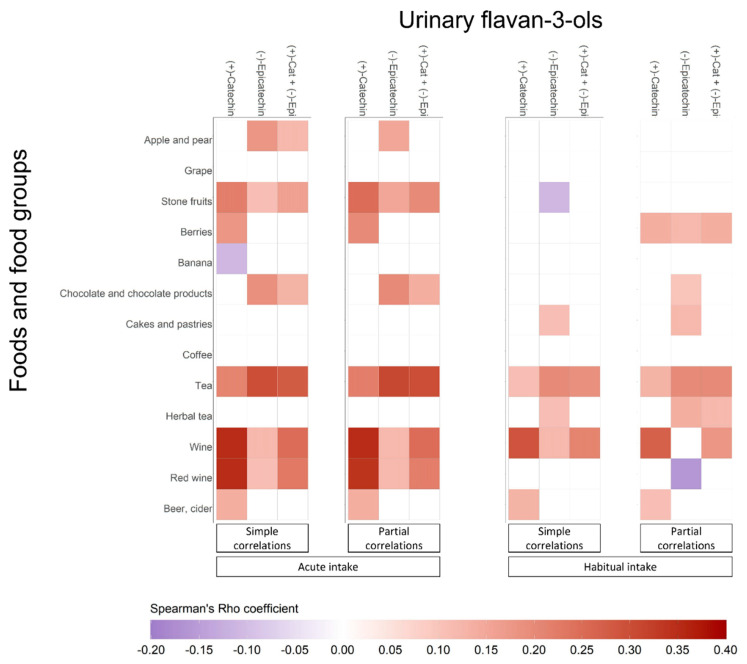
Heatmaps showing simple and partial Spearman’s correlations between urinary flavan-3-ols and intake (acute and habitual) of foods. Abbreviations: 24-HDR, 24-h dietary recall; (+)-Cat, (+)-catechin; DQ, dietary questionnaire; (-)-Epi, (-)-epicatechin.

Simple and partial Spearman’s correlations between urinary concentrations of flavan-3-ols and dietary flavan-3-ol-rich food sources are shown in Figure 2. Urinary concentrations of (+)-catechin, (-)-epicatechin and their sum correlated with acute and habitual intake of apple and pear, stone fruits, berries, banana, chocolate and chocolate products, cakes and pastries, tea, herbal tea, wine, red wine, and beer and cider. Partial correlations were found to be higher for acute than for habitual intakes. For acute intakes, the highest correlations were found between urinary (+)-catechin and the intake of wine (*r*_partial_ = 0.35, *p* < 0.001), red wine (*r*_partial_ = 0.34, *p* < 0.001), and between urinary (-)-epicatechin and the intake of tea (*r*_partial_ = 0.31, *p* < 0.001). Similarly, in habitual intakes the highest correlations were found between urinary (+)-catechin and wine (*r*_partial_ = 0.27, *p* < 0.001), and between (-)-epicatechin and tea (*r*_partial_ = 0.20, *p* < 0.001).

All simple and partial Spearman’s correlations, including rho (*r*) coefficients and statistical significance between urinary and dietary flavan-3-ols, and flavan-3-ol-rich food sources, according to their acute and habitual intakes are shown in Table S1.

## 4. Discussion

In the current study, we assessed the relationships between acute and habitual intake of flavan-3-ol monomers, proanthocyanidins and theaflavins, as well as of their main food sources, and 24-h urine concentrations of (+)-catechin and (-)-epicatechin in the EPIC study. In general, the urinary excretion of (+)-catechin, (-)-epicatechin and their sum were weakly-to-modestly correlated with total and individual intake of monomers, proanthocyanidins, and theaflavins, and with total intake of flavan-3-ols. All comparable correlations were stronger for acute than for habitual intakes, and also generally higher for urinary (-)-epicatechin than for urinary (+)-catechin. Notably, the majority of the observed correlations were similar after controlling for sociodemographic and lifestyle variables in the partial Spearman’s correlation analysis.

To our knowledge, only a few studies have assessed the use of urinary concentrations of (+)-catechin and (-)-epicatechin as potential nutritional biomarkers of flavan-3-ols. In our study, correlations between 24-h urine concentrations and acute intake of (+)-catechin and (-)-epicatechin were moderate (*r*_partial_ = 0.54 and 0.52, respectively). In a literature review of controlled intervention studies, Pérez-Jiménez, et al., showed a weak correlation between 24-h urine concentrations of (-)-epicatechin and its controlled dietary intake (*r* = 0.21), while no correlation data were presented for (+)-catechin [21]. Apart from being different study designs (i.e., observational vs. intervention), these differences in correlation coefficients between studies may be partially due to methodological aspects, such as differences in the methods used to estimate the intake and the urinary content of flavanol compounds, and the limited number of food or supplement sources used for the estimation of dietary intake.

As expected, concentrations of (+)-catechin, (-)-epicatechin and their sum in urine correlated with total and individual intake of monomers, ranging from *r*_partial_ = 0.40 to 0.49 (*p* < 0.001) and from *r*_partial_ = 0.22 to 0.28 (*p* < 0.001) for acute and habitual intakes of total flavan-3-ol monomers, respectively. Interestingly, these coefficients were found to be very similar to those observed with the acute (*r*_partial_ = 0.40–0.48, *p* < 0.001) and habitual intake (*r*_partial_ = 0.23–0.33, *p* < 0.001) of total dietary flavan-3-ols. This is because of a strong correlation between dietary intake of total monomers and total flavan-3-ols (r = 0.76, p < 0.001) for both acute and habitual intake was observed. In our study, acute and habitual intake of total flavan-3-ol monomers was lower than total proanthocyanidins, but higher than total theaflavins. This would mean that the single intake of flavan-3-ol monomers was not determining in the strength of the correlations observed.

In the current study, we also found weak but significant correlations between urinary concentrations of (+)-catechin, (-)-epicatechin and their sum, and individual and total intakes of proanthocyanidins and theaflavins. Specifically, acute intakes of total proanthocyanidins (*r*_partial_ = 0.32–0.38, *p* < 0.001), and both acute (*r*_partial_ = 0.22–0.30, *p* < 0.001) and habitual intake (*r*_partial_ = 0.20–0.25, *p* < 0.01) of theaflavins correlated with the urinary excretion of (+)-catechin, (-)-epicatechin and their sum. Meanwhile, habitual intake of total proanthocyanidins only correlated with the sum of (+)-catechin and (-)-epicatechin (*r*_partial_ = 0.22–0.30, *p* < 0.05). Proanthocyanidins dimers and trimers are poorly absorbed (5–10% of (-)-epicatechin). They are conjugated by Phase II enzymes, and scarcely depolymerized to monomers [22,23]. Indeed, proanthocyanidins (specially >tetramers) all reach the colon where they are transformed by the intestinal microbiota before absorption as small phenolic acids [6]. Consequently, proanthocyanidins do not largely contribute to the concentration of flavan-3-ol monomers in blood and urine. Studies on bioavailability of theaflavins are scant. Recently, in an acute intervention study, where participants ingested 1 g supplement containing 998 μmol of a mixture of theaflavins, the estimated 0–30 h bioavailability of the mixture of theaflavins was insignificant (<0.000001%) [24]. Similar to other flavan-3-ols, a large proportion of theaflavins is metabolized by colonic microbiota into phenolic acids, phenyl-γ-valerolactones, phenyl-γ-hydroxyvaleric acids, and their free and conjugated forms [24,25]. To the best of our knowledge, there are no previous data showing that theaflavins can be depolymerized in the small intestine into flavan-3-ol monomers.

Altogether, the data from our study show that neither the acute nor habitual intake of proanthocyanidins and theaflavins contributed notably to the urinary concentrations of (+)-catechin and (-)-epicatechin. Thus, the presence of both free and conjugated forms of (+)-catechin and (-)-epicatechin compounds in urine would be more suitable indicators of the intake of individual and total flavan-3-ol monomers of food sources that contain flavan-3-ol monomers and proanthocyanidins and/or theaflavins together. Indeed, Ottaviani, et al., recently found that the 24 h urinary excretion of three structurally related (-)-epicatechin metabolites, namely (-)-epicatechin-3′-glucuronide, (-)-epicatechin-3′-sulfate and 3′-O-methyl-(-)-epicatechin-5-sulfate, was correlated with the acute dietary intake of (-)-epicatechin but not with procyanidin B2, thearubigins and theaflavins [26].

A growing number of studies suggest that instead of intact or native flavan-3-ol compounds, some of their derived microbial metabolites named hydroxyphenyl-γ-valerolactones and hydroxyphenyl-γ-valeric acids could be used as better indicators of individual and total intake of flavan-3-ols, particularly for monomers and dimers [22,27,28]. The specificity of 5-(3′,4′-dihydroxyphenyl)-γ-valerolactone as a biomarker of dietary flavan-3-ol monomers and dimers was corroborated in a study where a single oral intake of (-)-epicatechin, (-)-epicatechin-3-O-gallate and procyanidin B-2 resulted in 24 h urine excretions of both 5-(3′,4′-dihydroxyphenyl)-γ-valerolactone-(3′/4′-sulfate) and 5-(3′,4′-dihydroxyphenyl)-γ-valerolactone-(3′/4′-O-glucuronide) [27]. However, the consumption of theaflavins, thearubigins, (-)-epigallocatechin and (-)-epigallocatechin-3-O-gallate, did not result in the formation of 5-(3′,4′-dihydroxyphenyl)-γ-valerolactone aglycone or Phase II metabolites in urine. These findings were similar to the found made by Hollands, et al., who reported that the 24 h urinary excretion of total hydroxyphenyl-γ-valerolactones was tenfold higher after the chronic intake of a high dose of (-)-epicatechin than after the chronic intake of procyanidins dimers-decamers [29]. In our study, free and Phase-II-conjugates of hydroxyphenyl-γ-valerolactones were not determined due to the lack of standard compounds warranted for their acute quantification. We believe that the inclusion of these microbial metabolites in future studies investigating flavan-3-ol biomarkers would improve the correlations observed here. Consistently with our hypothesis, Ottaviani, et al., recently showed that the sum of 24-h urinary excretions of 5-(3′/4′-dihydroxyphenyl)-γ-valerolactone-3′/4′-sulphate and O–glucuronide metabolites was strongly and consistently correlated (Spearman’s r = 0.90; Pearson’s r = 0.81) with total intake of flavan-3-ols in an acute intervention study [27]. Urinary (-)-epicatechin was found more strongly correlated with intake of total monomers and total flavan-3-ols, as well as with total and individual intake of proanthocyanidins and theaflavins than urinary (+)-catechin. This finding was expected for two main reasons: (i) the higher dietary intake (both acute and habitual) of (-)-epicatechin than (+)-catechin among participants; and (ii) the higher intestinal absorption of (-)-epicatechin compared with (+)-catechin [6].

Weak but significant correlations were observed between urinary (+)-catechin and (-)-epicatechin concentrations and the intake of apple and pear, stone fruits, berries, chocolate and chocolate products, cakes and pastries, tea, herbal tea, wine, red wine, and beer and cider. These correlations would be consistent with previous studies showing the presence of (+)-catechin and/or (-)-epicatechin metabolites in human urine and plasma after the consumption of the mentioned foods. Apple and pear are rich-sources of flavan-3-ols, particularly proanthocyanidins. Regarding monomers, (-)-epicatechin compounds are found in higher concentrations than (+)-catechin in both apples and pears [30]. Furthermore, urinary excretion of (-)-epicatechin metabolites, but not (+)-catechin, has been extensively reported in controlled dietary intervention studies with apples [31]. Apricot, peach, plum and nectarine are sources of (+)-catechin and (-)-epicatechin [32,33], but, to our knowledge, they were not previously correlated with urinary flavanol-3-ol concentrations. Acute intake of berries was only correlated with urinary (+)-catechin, whereas their habitual intake correlated with both urinary (+)-catechin and (-)-epicatechin and their sum. Berries are sources of flavan-3-ol monomers, especially (+)-catechin [34], which would explain the higher correlations observed between the urinary concentrations with this compound. Acute and habitual intake of chocolate and chocolate products was weakly correlated with urinary (-)-epicatechin. (+)-catechin, (-)-catechin and (-)-epicatechin derivatives are by far the most reported group of metabolites after cocoa intake, followed by hydroxyphenyl-γ-valerolactones, phenyl-γ-hydroxyvaleric acids and methylxanthines [35]. However, it was recently found in an intervention study that the appearance of (-)-epicatechin in plasma was higher than (±)-catechin after cocoa consumption [36], suggesting a lower bioavailability of catechin enantiomers. Habitual but not acute intake of cakes and pastries was weakly but significantly correlated with urinary (-)-epicatechin concentrations. This finding is not surprising, first because the habitual intake was higher than the acute one; and second because most bakery products are usually made with flavan-3-ol-rich ingredients, including cocoa, berries, and fruits [37]. Such as in our study, urinary excretion of (+)-catechin and (-)-epicatechin metabolites has been largely reported after tea consumption in controlled intervention trials and correlated with their intake in observational studies [38]. All comparable correlations were higher for urinary (-)-epicatechin than for (+)-catechin, also suggesting the lower bioavailability of catechin. Furthermore, higher correlations with acute than habitual intake of tea could be due to urinary biomarkers better reflecting short-term rather than long-term exposure [39]. Herbal tea comprises a long list of beverages made from infusion or decoction of stems, leaves and other parts of one or more plants in hot water. This beverage is rich in phenolic compounds, including flavan-3-ols, which would make the observed correlations expectable between the (habitual) intake of herbal tea and urinary concentrations of (-)-epicatechin and its sum with urinary (+)-catechin. In our study, we found that the acute intakes of both wine and red wine were similarly correlated with urinary flavan-3-ols. Red wine is consumed more and contains higher amounts of flavan-3-ol compounds than white and rosé wines [40,41]. The weak but significant correlation between urinary (+)-catechin and the intake (acute and habitual) of beer and cider observed in this study is in line with previous studies, showing that (+)-catechin and (+)-catechin compounds are some of the most abundant polyphenols found in beer [42] and cider [43].

The strengths of our study include the availability of data on acute and habitual food intake among a relative high sample size of participants of the EPIC study, also the availability of 24-h urine samples, which offers additional advantages for the accurate assessment of polyphenol metabolites over both spot urine and plasma samples [44]. Another strength is the high sensitivity of the analytic method used to measure the urinary concentrations of (+)-catechin and (-)-epicatechin. Our study also presents some limitations. First, our results could be influenced by random and systematic errors in the food intake assessment; however, both the DQ and the 24-HDR used were center/country validated [16,45]. Second, the real estimation of flavan-3-ol intake was likely to be underestimated due to possible foods with unknown flavan-3-ol data composition, although the Phenol-Explorer database is one of the most comprehensive databases on flavan-3-ols [46]. Another potential limitation is that our study did not contemplate the inclusion of urinary microbial metabolites derived from flavan-3-ol intake (e.g., hydroxyphenyl-γ-valerolactones). In future studies investigating flavan-3-ol biomarkers would be essential to evaluate the use of derived microbial metabolites, instead of parent or conjugated compounds, especially as nutritional biomarkers of proanthocyanidins and theaflavins.

## 5. Conclusions

In conclusion, 24-h urinary excretion of (+)-catechin, (-)-epicatechin, and their sum was moderately and weakly correlated with the acute and habitual intake of flavan-3-ols, respectively, particularly with total flavan-3-ol monomers. Therefore, urine (+)-catechin and (-)-epicatechin and their sum can be considered as moderate biomarkers of acute intake of flavan-3-ol monomers. Urinary flavan-3-ol concentrations correlated poorly with proanthocyanidins and theaflavins, making them, therefore, not useful as nutritional biomarkers for these subgroups of flavan-3-ols. Since proanthocyanidins and theaflavins are poorly metabolized into monomers, the low correlations between these and urinary (+)-catechin and (-)-epicatechin are due to the intake of some shared food sources between all flavan-3-ols, such as tea.

## Figures and Tables

**Table 1:** Medians, and 10th (P10) and 90th (P90) percentiles of urinary excretion (μmol/24 h) of (+)-catechin, (-)-epicatechin, and their sum according to sociodemographic and lifestyle characteristics in a subsample (*n* = 419) of the European Prospective Investigation into Cancer and Nutrition (EPIC) study.

		(+)-Catechin				(-)-Epicatechin				Sum of (+)-Catechin + (-)-Epicatechin		
Characteristics	*N* ^a^	*n* ^b^	Median	P10	P90	*n* ^b^	Median	P10	P90	Median	P10	P90
All	419	22	0.12	0.03	0.39	18	0.23	0.08	0.58	0.34	0.12	0.93
Center												
Paris (FRA)	67	2	0.14	0.05	0.41	2	0.28	0.10	0.61	0.45	0.17	0.97
Florence (ITA)	45	3	0.10	0.02	0.26	3	0.18	0.05	0.34	0.25	0.07	0.55
Varese (ITA)	51	2	0.13	0.03	0.31	3	0.21	0.10	0.45	0.33	0.12	0.74
Ragusa (ITA)	17	1	0.08	0.03	0.25	3	0.19	0.08	0.35	0.27	0.05	0.51
Turin (ITA)	42	0	0.15	0.04	0.67	2	0.25	0.10	0.61	0.45	0.17	1.47
Naples (ITA)	20	1	0.06	0.03	0.39	0	0.17	0.06	0.36	0.20	0.09	0.63
Heidelberg (GER)	59	7	0.15	0.04	0.48	1	0.29	0.09	0.87	0.41	0.10	1.18
Potsdam (GER)	118	6	0.10	0.03	0.36	4	0.26	0.10	0.58	0.35	0.15	0.85
Sex												
Men	171	10	0.15	0.04	0.42	5	0.27	0.10	0.61	0.40	0.16	1.05
Women	248	12	0.10	0.03	0.38	13	0.21	0.08	0.53	0.32	0.11	0.86
Age (y)												
<50	135	6	0.10	0.03	0.37	6	0.22	0.08	0.63	0.32	0.12	0.98
50–60	188	8	0.12	0.03	0.42	7	0.23	0.08	0.58	0.36	0.12	0.94
>60	96	8	0.12	0.04	0.42	5	0.23	0.12	0.56	0.35	0.16	0.85
BMI (kg/m^2^)												
<25	201	15	0.12	0.03	0.39	10	0.21	0.08	0.58	0.34	0.10	0.90
25 to <30	160	5	0.12	0.04	0.43	7	0.25	0.09	0.59	0.37	0.15	0.98
≥30	58	2	0.09	0.04	0.33	1	0.23	0.09	0.57	0.32	0.14	0.88
Smoking status ^c^												
Never smoked	211	12	0.11	0.03	0.36	9	0.23	0.09	0.61	0.33	0.13	0.95
Former smoker	120	4	0.15	0.03	0.42	5	0.23	0.09	0.53	0.37	0.12	0.89
Current smoker	78	6	0.10	0.03	0.36	3	0.20	0.06	0.51	0.29	0.08	0.80
24-HDR total energy intake (kcal/day)												
<1750	112	2	0.11	0.03	0.41	4	0.23	0.08	0.53	0.33	0.13	0.92
1750–2375	156	11	0.11	0.03	0.37	10	0.21	0.08	0.57	0.32	0.10	0.88
>2375	151	9	0.13	0.04	0.36	4	0.24	0.10	0.60	0.37	0.15	0.99

^a^ Number of total participants (including consumers and non-consumers) within the corresponding category; ^b^ subjects <LOQ. ^c^ Data from ten participants were not available. Abbreviations: 24-HDR, 24-h dietary recall, FRA, France, ITA, Italy, GER, Germany.

**Table 2:** Medians, and 10th (P10) and 90th (P90) percentiles of dietary intakes of flavan-3-ols, glycosides and aglycones and their main food sources in the European Prospective Investigation into Cancer and Nutrition (EPIC) study.

Variable	Acute Intake (24-HDR)				Habitual Intake (DQ)			
Flavanols Intake (mg/day)	*n* ^a^	Median	P10	P90	*n* ^a^	Median	P10	P90
Total flavan-3-ols	2	833	81.2	2374	0	317	156	627
Total monomers	2	106	21.0	685	0	54.1	18.6	249
(+)-Catechin	2	29.8	7.03	103	0	14.0	5.59	31.8
(+)-Catechin 3-O-gallate	222	0.00	0.00	41.9	16	0.90	0.00	17.8
(+)-Catechin 3-O-glucose	408	0.00	0.00	0.00	45	0.05	0.01	0.44
(+)-Gallocatechin	85	0.17	0.00	189	0	2.50	0.03	47.2
(+)-Gallocatechin 3-O-gallate	225	0.00	0.00	6.76	7	0.19	0.00	2.82
(-)-Epicatechin	2	50.5	6.07	151	0	18.1	7.61	38.5
(-)-Epicatechin 3-O-gallate	41	3.44	0.00	78.3	0	3.58	0.37	33.0
(-)-Epigallocatechin	63	0.52	0.00	109	0	1.99	0.06	31.4
(-)-Epigallocatechin 3-O-gallate	205	0.06	0.00	93.4	1	2.55	0.01	39.8
(-)-Ec-Ec	276	0.00	0.00	1.61	9	0.09	0.02	0.40
Total PA	6	576	46.7	1723	0	216	98.7	427
PA dimers	6	106	9.56	355	0	22.1	9.44	44.8
PA trimers	10	48.5	5.18	171	0	18.2	7.65	36.7
PA tetramers-hexamers	33	127	0.84	382	0	50.3	22.3	101
PA heptamers-decamers	43	90.7	0.00	293	0	35.0	16.4	73.9
PA > decamers	45	176	0.00	624	0	87.1	41.8	168
Total theaflavins	314	0.00	0.00	135	133	8.93	0.50	56.8
Theaflavin	314	0.00	0.00	44.1	133	2.34	0.13	14.9
Theaflavin 3,3′-O-digallate	314	0.00	0.00	30.9	133	2.53	0.14	16.1
Theaflavin 3′-O-gallate	314	0.00	0.00	43.3	133	2.92	0.16	18.6
Theaflavin 3-O-gallate	314	0.00	0.00	16.8	133	1.13	0.06	7.21
Food intake (g/day)								
Apple and pear	222	0.00	0.00	374	16	51.8	6.40	182
Grape	402	0.00	0.00	0.00	26	7.14	0.27	27.7
Stone fruits	342	0.00	0.00	126	15	36.8	3.75	117
Berries	381	0.00	0.00	0.00	24	7.14	0.41	28.6
Banana	356	0.00	0.00	94.5	84	7.21	0.00	38.6
Chocolate and chocolate products	313	0.00	0.00	27.0	94	3.28	0.00	20.0
Cakes and pastries	243	0.00	0.00	160	20	30.1	3.29	102
Coffee	53	175	0.00	769	29	150	17.1	600
Tea	310	0.00	0.00	500	133	21.2	0.00	436
Herbal tea	327	0.00	0.00	417	268	0.00	0.00	160
Wine	234	0.00	0.00	375	51	55.3	0.00	357
Red wine	303	0.00	0.00	267	291	0.00	0.00	222
Beer and cider	355	0.00	0.00	324	133	5.52	0.00	283

^a^ Number of non-consumers; abbreviations: 24-HDR, 24-h dietary recall; DQ, dietary questionnaire; Ec-Ec, (-)-epicatechin-(2a-7)(4a-8)-epicatechin 3-O-galactoside; PA, proanthocyanidins.

## Data Availability

The data presented in this study are available on request from the corresponding author.

## Supplementary Materials

The following are available online at https://www.mdpi.com/2072-6643/13/11/4157/s1, Table S1: simple and partial Spearman’s correlation coefficients between 24-h urinary concentrations of (+)-catechin and (-)-epicatechin and intake (acute and habitual) of flavan-3-ols, and foods.
